# 30-Day Outcomes after Surgical or Transapical Aortic Valve Replacement in Symptomatic Aortic Regurgitation

**DOI:** 10.3390/jcdd9070224

**Published:** 2022-07-14

**Authors:** Minjian Kong, Ze Hong, Xianbao Liu, Xian Zhu, Jianan Wang, Aiqiang Dong

**Affiliations:** 1 Department of Cardiac and Macrovascular Surgery, Second Affiliated Hospital of Zhejiang University School of Medicine, Hangzhou 310009, China; kmj@zju.edu.cn (M.K.); dr_hongze@163.com (Z.H.); dr_zhuxian@zju.edu.cn (X.Z.); 2 Department of Cardiology, Second Affiliated Hospital of Zhejiang University School of Medicine, Hangzhou 310009, China; liuxb@zju.edu.cn (X.L.); wangjianan111@zju.edu.cn (J.W.)

**Keywords:** aortic regurgitation, transapical aortic valve replacement, J-valve™ system, surgical aortic valve replacement, propensity score matching

## Abstract

**Background**: We aimed to analyze the short-term clinical outcomes of transapical aortic valve replacement (TA-TAVR) compared with surgical aortic valve replacement (SAVR) in symptomatic aortic regurgitation (AR) patients to draw preliminary conclusions about the advantages and disadvantages of TA-TAVR compared with SAVR and to provide evidence for future use of TA-TAVR in AR patients. **Method**: From September 2016 to September 2021, 69 patients undergoing TA-TAVR with J-valve implantation and 42 patients undergoing SAVR at the Second Hospital of Zhejiang University School of Medicine were analyzed for clinical data and 30-day follow-up outcomes to analyze and compare the differences in clinical endpoints between the two procedures. **Results**: At 30-day follow-up, there were no significant differences in mortality or neurological events between the two groups before and after the PSM. In secondary endpoints there were significant differences between the pre-match TAVR and SAVR groups, such as the incidence of paravalvular leaks (33.8% vs. 4.8%, *p* < 0.05), which also remained after the PSM (37.5% vs. 0, *p* < 0.05). In addition, the incidence of major bleeding was 7.4% in the TAVR group and 26.2% in the SAVR group before matching (*p* < 0.05). After matching, the statistical difference still remained. In longitudinal comparison, significant improvements in postoperative cardiac ultrasound indices and NYHA classification occurred in both groups. **Conclusion**: The TA-TAVR approach is safe and reliable, with similar clinical efficacy to SAVR, and has advantages in bleeding rate and speed of recovery.

## 1. Introduction

Since the invention of transcatheter aortic valve replacement in 2002, TAVR has gradually become the treatment of choice for patients with moderate to high risk of surgical contraindicated aortic valve disease as clinical trials have continued to progress and relevant clinical outcomes have continued to be discovered [1,2]. As demand increases, the efficacy and benefit of TAVR in different risk groups need to be further confirmed. Several preliminary results have been obtained from a number of the clinical trials, and these results are exciting [3,4,5,6,7,8]. It is foreseeable that TAVR may become an optional treatment in the future alongside surgery, regardless of the patient’s surgical risk.

There are several approaches to TAVR, with the transfemoral approach being the most common. The transapical approach expands the population for which TAVR is indicated as an alternative means to access to the periphery due to anatomy or disease [9]. Although the transapical approach is more damaging than other peripheral approaches, many studies have validated its effectiveness and safety [10,11,12]. There is still a general consensus that aortic regurgitation is not on-label for TAVR, by either transfemoral or transapical approach [13]. The main reason for this is the unique pathological mechanism of AR. The anatomy of AR patients reveals that their valves are rarely calcified, and the aortic annulus is often dilated, which makes it difficult to anchor the first-generation valves and some of the second-generation valves to their intended positions by conventional means, leading to the common occurrence of secondary valve implantation, valve dislocation, annular rupture, residual regurgitation, and paravalvular leakage after surgery [14]. To overcome the problem, physicians and researchers have conducted numerous experiments on and made improvements to the valve and positioning release device. The J-valve™ system, with its unique positioning mechanism and valve anchoring, has broken through the limitation of TAVR in patients with purely non-calcified AR, which can also be performed through a transapical pathway. A large number of clinical trials have been performed to validate its safety and short- to medium-term efficacy [15,16,17,18].

In order to further validate the efficacy of TAVR and evaluate the advantages and disadvantages of TAVR and surgery, numerous clinical trials had been conducted at home and abroad, and the medium and long-term effects of some valves have been demonstrated [7,8,19]. Although it is now clear that TAVR is a first-line treatment option for aortic valve disease patients at moderate to high surgical risk, until the advent of second-generation valves such as J-valve, these clinical trials tended to be conducted in patients with aortic stenosis; and few studies have compared the efficacy, advantages, and disadvantages of TAVR and SAVR in patients with pure AR. In this article, we will collect and analyze data on the safety and efficacy of TAVR with the J-valve™ system compared with surgical placement of bioprosthetic valves in patients with pure or predominantly aortic valve regurgitation so as to provide a preliminary description of the advantages and disadvantages of TAVR compared to SAVR.

## 2. Materials and Methods

From September 2016 to September 2021, clinical and follow-up data were retrospectively and consecutively collected on 69 patients with pure or predominant aortic regurgitation who had transapical pathway valve replacement with the J-valve™ system. Inclusion criteria for patients in the TAVR group: (1) Aortic regurgitation assessed as requiring surgical intervention according to ESC/EACTS guidelines [2]; (2) High surgical risk as assessed by the cardiac team. (3) Difficulty with peripheral access. Exclusion criteria: (1) patients with life expectancy <1 year; (2) contrast allergic reactions; (3) inappropriate anatomical conditions. At the same time, 298 patients who underwent simple aortic valve replacement were found in the surgical database, and 42 patients remained after excluding patients with pure or predominant aortic stenosis, secondary surgery, and implantation of mechanical valves. All of the studies were reviewed and approved by the Ethics Committee of the Second Hospital of Zhejiang University School of Medicine.

Baseline information collected included age; gender; BMI; STS score; previous underlying diseases, such as hypertension and diabetes; and left ventricular ejection fraction. The 30-day follow-up primary endpoints and adverse event reports for patients in the TAVR and SAVR groups were formulated according to the VARC-2 guidelines [20]. The primary postoperative endpoints were success of valve implantation and in-hospital all-cause mortality. Secondary endpoints were length of hospital stay and procedure-related bleeding rate. The primary endpoints at 30-day follow-up were all-cause mortality and paravalvular leakage. Secondary endpoints were rehospitalization rate, new cerebrovascular accidents, atrial fibrillation, and myocardial infarction. Given the different criteria for judging bleeding in surgical and interventional procedures, this paper mainly refers to the bleeding criteria in the article by Philippe Généreux et al. [21]. Major bleeding was defined, with appropriate modifications, as meeting any of the following criteria along with having a clear site of bleeding: (1) bleeding that caused death; (2) bleeding that caused a new hospitalization; (3) bleeding that required pericardiocentesis or open and/or endovascular procedure for repair or hemostasis; (4) bleeding that caused permanent disability (e.g., blindness, paralysis, and hearing loss); and (5) bleeding that required transfusion of ≥3 U of blood within a 24 h period. Furthermore, taking into account the effect of discrepancies between the two groups of baseline data on the results, the propensity score matching was performed on 20 indicators from the baseline data using the IBM SPSS Statistics 26 statistical package, yielding a total of 16 pairs of matched results, which were analyzed again.

Data were processed using IBM SPSS Statistics 26 data statistical package for statistical analysis. The measurement data were expressed as mean ± standard deviation, and the independent sample t-test and rank sum test were used for comparison between groups, and paired *t*-test was used for comparison of data within groups. Count data were expressed as frequencies, and the χ^2^ test was used for comparison between groups. Differences were considered statistically significant at *p* < 0.05.

## 3. Results

### 3.1. Baseline Data

In this study, 69 patients with moderate to severe aortic regurgitation who underwent TA-TAVR were retrospectively included from September 2016 to September 2021, in addition to 42 patients with the same diagnosis who underwent simple aortic valve bioprosthesis replacement during this timeframe screened from the surgical database. Table 1 mainly describes the baseline data of the TAVR and SAVR groups before and after propensity matching, from which it can be seen that the main differences between the two groups before matching were the STS score (1.48 ± 1.73% vs. 3.76 ± 3.93%, *p* < 0.05), the incidence of atrial fibrillation disease history (26.1% vs. 4.8%, *p* < 0.05), and LVEF (50.84 ± 12.38% vs. 57.30 ± 12.13%, *p* < 0.05). In relation to the reasons for these variations, it was mainly considered that patients in the TAVR group had a worse underlying condition compared to the SAVR group; that is why the cardiac team recommended them to opt for TAVR. The baseline of patients in both groups after matching was essentially the same.

### 3.2. Surgery

All of the patients in the TAVR group were operated on under general anesthesia. Except for one case in which the valve was severely displaced intraoperatively and converted to open chest, all of the patients were successfully implanted with J-VALVE. The implanted valves were mainly over 27 mm in size (85.9%), and no significant prosthetic regurgitation was observed on transesophageal echocardiography intraoperatively. In the SAVR group, all patients were operated with cardiac pulmonary bypass under general anesthesia, the patients were successfully implanted with prosthetic valves of 25 mm or larger (64.3%), and all patients were evaluated by intraoperative transesophageal echocardiography. No significant prosthetic regurgitation was observed. No perioperative deaths occurred in either group.

### 3.3. 30-Day Follow-Up

**Figure 1:**
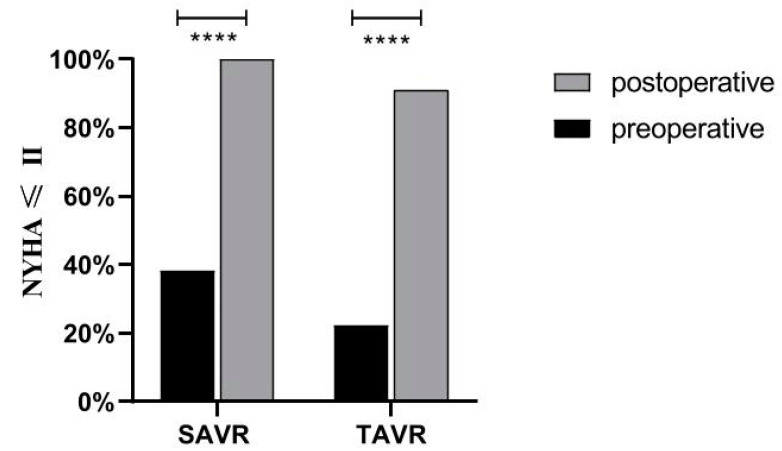
NYHA classification in both groups before and after procedure. **** means *p* ≤ 0.0001.

The primary and secondary endpoint results at 30-day follow-up for the TAVR and SAVR groups before and after matching are described detailed in Table 2. Neither group had a significant difference in mortality before or after matching. In the secondary endpoints, the differences were mainly in the speed of recovery, paravalvular leak incidence, and rate of bleeding. This was reflected in the data as the mean total hospital stay, and ICU stay in the TAVR group was significantly shorter than in the SAVR group (before matching: TAVR 11.41 ± 10.43 days vs. SAVR 11.29 ± 3.73 days, *p* < 0.05; TAVR 1.86 ± 5.78 day vs. SAVR 5.05 ± 2.02 days, *p* < 0.05; after matching: TAVR 12.37 ± 13.79 days vs. SAVR 12.63 ± 4.44 days, *p* < 0.05; TAVR 3.50 ± 10.83 day vs. SAVR 5.88 ± 2.60 days, *p* < 0.05). There was better performance in the TAVR group than in the SAVR group in terms of the major bleeding (before matching: TAVR 7.4% vs. SAVR 26.2%, *p* < 0.05; after matching: TAVR 6.3% vs. SAVR 37.5%, *p* < 0.05). However, for paravalvular leaks, two patients with paravalvular leaks were detected at 30-day follow-up in the SAVR group; both cases were mild. Twenty-three patients with paravalvular leaks were seen in the TAVR group, 19 with mild and 4 with moderate. No patients had severe paravalvular leaks, and there was a statistical difference between the two groups (before matching: TAVR 33.8% vs. SAVR 4.8%, *p* < 0.05; after matching. (TAVR 37.5% vs. SAVR 0). In relation to the incidence of the 3rd degree AV block and the rate of permanent pacemaker (PPM) implantation, there were six patients with 3rd degree AV block in the TAVR group before matching, and five of them had PPM implantation. No patients in the SAVR group were found to have 3rd degree AV block, nor did any patients have PPM implantation for other reasons. Finally, for an objective evaluation of the efficacy of the procedures in both groups, Figure 1 shows the change in NYHA classification of patients both groups before and after surgery, which shows that the percentage of patients with NYHA grade III or higher decreased significantly in both groups postoperatively.

### 3.4. 30-Day Cardiac Ultrasound

**Figure 2:**
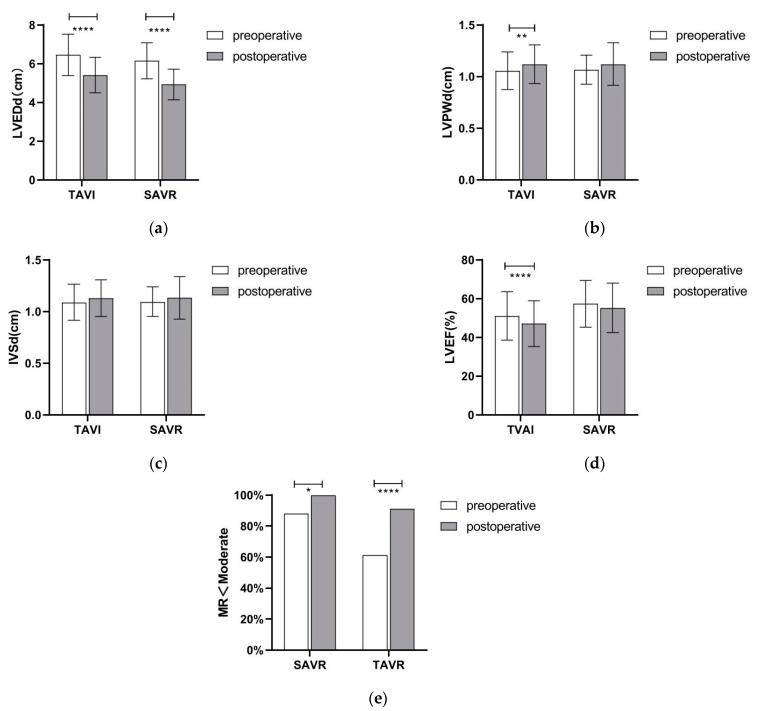
Cardiac ultrasound data changes in both groups before surgery and at 30-day follow-up: (**a**) LVEDd, left ventricular end-diastolic diameter; (**b**) LVPWd. left ventricle posterior wall daimeter; (**c**) IVSd, interventricular Septal diameter; (**d**) LVEF, left ventricular ejection fraction; (**e**) MR, mitral valve regurgitation. * means *p* ≤ 0.05, ** means *p* ≤ 0.001, and **** means *p* ≤ 0.0001.

Figure 2 describes the preoperative and 30-day follow-up echocardiographic data of both groups, which show a significant improvement in left ventricular diameter in both the SAVR and TAVR groups pre- and post-intervention. Furthermore, the preoperative rate of patients with combined moderate or greater mitral valve insufficiency decreased significantly after both procedure. Whether the patients were in the SAVR or TAVR group, at the 30-day follow-up, LVEF showed some degree of decrease, even though clinical symptoms improved in both groups.

## 4. Discussion

Since 2002, the TAVR procedure has quickly become one of the prominent clinical options for the treatment of aortic valve-related disease [22]. In past years, with continuous improvements in procedures and valve devices and the performance of various clinical trials, the TAVR procedure is narrowing its limitations in patients with various types of aortic valve lesions, but its constraints in patients with aortic regurgitation are still one of the topical issues of ongoing research [13]. The J-valve™ system, a new generation valve device launched in 2014 [23], was originally developed to address the limitations of TAVR when treating patients with aortic regurgitation with no calcification and an enlarged annulus. Numerous studies have been conducted to verify its safety and short- and medium-term efficacy after the CFDA approval to market it [15,16,17,18]. Such studies, however, were often conducted solely to evaluate the efficacy of the J-valve in terms of improvement in symptoms and clinical indicators before and after surgery, and the incidence of complications such as paravalvular leaks, bleeding, and new cerebrovascular accidents, whereas few articles described comparative studies with surgically treated patients.

The baseline data, surgery-related data, and 30-day follow-up results of 69 patients with symptomatic moderate to severe aortic regurgitation were retrospectively collected according to inclusion and exclusion criteria, and 42 surgical patients were retrospectively collected and filtered for comparison of outcomes. The main reason for not specifying a particular risk stratum in the initial data collection is that more and more TAVR studies have already transitioned to low-risk groups from medium- to high-risk groups, and preliminary trials in low-risk groups have shown promising short-term and medium-term efficacy of TAVR [3,4,5], so we aimed to comprehensively evaluate the efficacy of TAVR in each risk stratum and expect to guide the choice of treatment modality for patients in different risk groups in the future. Since some differences existed in the baseline data between the two groups, propensity score matching was also performed to ensure accuracy of the relevant results.

Similarly to previous studies comparing surgical and interventional procedures [6,7,21,24], the primary and secondary endpoints of postoperative all-cause mortality, incidence of cerebrovascular accidents, and incidences of different degrees of paravalvular leakage were evaluated in the two groups according to the criteria of the VARC-2 in the current study. The results showed that the two groups exhibited significant differences in length of hospital stay, ICU stay, and incidence of paravalvular leakage before and after propensity score matching. It has been demonstrated in earlier studies of TA-TAVR that transapical interventions can also effectively reduce the ICU length of stay and total length of hospital stay in patients compared to surgical procedures [11,25], and the findings of this study are similar to the above studies. The difference between the TAVR group and the SAVR group in terms of paravalvular leakage was mainly in the mild paravalvular leakage, which did not have a significant impact on the overall survival or quality of life in terms of short-term outcomes [26]. The incidence of paravalvular leakage greater than moderate was 5.9% before matching and 6.3% after matching in the TAVR group, both well below the results in similar studies [27].

The most common disadvantages of surgical procedures compared to interventional procedures are greater trauma, a higher transfusion rate, and more bleeding. TA-TAVR, as one of the more traumatic branches of interventional procedures, has few known advantages compared to surgical procedures, mainly because the bleeding in surgical procedures differs significantly from that of interventional bleeding as defined by the VARC-2 guidelines. A more scientific standard of comparison needs to be developed by the cardiac team if such a comparison is to be made. In this paper, we refer to the criteria of Philippe Généreux et al. for comparing the degree of postoperative bleeding trauma in two groups [21]. According to the criteria, TA-TAVR, the more invasive of the interventional means, also has a much lower incidence of major bleeding than surgical procedures, whether before or after matching, whose safety in the primary observation endpoint of bleeding is reliable.

Nowadays, it is generally assumed that AV block caused by TAVR is clearly related to the anatomical characteristics of the aortic root. Below the right coronary valve and the noncoronary valve is the septal membrane part, which contains the atrioventricular bundle penetration branch and the left bundle branch, both of which are very superficially located under the endocardium, where the AV block or left bundle branch conduction block will occur with slight compression or injury. These conduction bundle problems may occur if the prosthesis is placed too deeply into the left ventricular outflow tract during earlier TAVR or if the prosthetic stent provides excessive radial force during valve anchoring. Our study showed that the incidence of third-degree AV block was only 8.8% after TAVR implantation of the J-valve, whereas it was only 7.4% for implantation of the PPM, significantly less than in other previous studies of transapical or transfemoral procedures [28,29,30,31]. This was attributed to the special anchoring mechanism of the J-valve. When anchoring is performed, the three U-shaped grippers on the outside of the stent combine with the metal stent to hold the diseased leaflet firmly in place, which reduces the need for radial force during valve fixation, which can effectively prevent the occurrence of the above two points [32], resulting in a lower prevalence of a third-degree AV block. However, the incidence was still higher than in the surgical group of our study, albeit without significant differences. Thus, even though the anchoring of the valve and the depth of valve implantation have been improved, further improvements in the incidence of arrhythmic complications, such as third-degree AV block after TA-TAVR using the J-valve™ system, remain to be studied in more detail. Interestingly, before propensity matching, the incidence of new-onset AF was much higher in the TAVR group than the SAVR group (16.2% vs. 2.4%, *p* < 0.05), but the difference disappeared after matching (12.5% vs. 6.3%, *p* = 0.54). We consider that an explanation for this change is that the two groups were much closer to each other at baseline after matching. The occurrence of atrial fibrillation was associated with age, obesity, and systemic inflammation levels [33].

Aortic regurgitation represents a compensatory pathophysiologic change in left ventricular remodeling, increased left ventricular size and volume, and left ventricular wall thinning [34,35]. To explore the improvements in objective indicators of cardiac function postoperatively, transthoracic echocardiography was performed before and 30 days after surgery in both groups, which showed that left ventricular reverse remodeling (left ventricular volume, volume reduction, left ventricular posterior wall, and septal thickening) happened in both groups by 30 days. As the left ventricular remodeling led to an improvement in cardiac function and a decrease in mitral regurgitation, whether aortic valve surgery requires co-treatment of mitral valve problems would be a matter of meticulous preoperative judgment. In both groups, the postoperative LVEF dropped to a certain extent, mainly due to the procedure strike, which could not be neutralized by recovery during the short-term follow-up. In addition, due to excessive regurgitation, higher output per beat and faster heart rate were required to compensate for the effects of regurgitation, resulting in overestimated LVEF, leading to clinical symptoms of fatigue, shortness of breath, and a decrease in activity endurance. After the procedure, the regurgitation problem was resolved, so that the compensatory effects of increased stroke volume and heart rate were reduced. LEVF was more reflective of left ventricular function, so the clinical symptoms improved postoperatively, despite the decrease in LVEF.

Finally, for the patients’ subjective index NYHA classification, we observed a significant improvement in postoperative symptom complaints, activity tolerance in both groups, and a significant decrease in the proportion of patients with NYHA grade III or higher.

## 5. Conclusions

According to current guidelines for the treatment of aortic regurgitation, surgery remains the preferred treatment option in patients for whom surgical indications exist and who can tolerate it [1,36]. This study adequately demonstrates the safety and short-term reliable efficacy of transapical TAVR implantation of J-valve in patients with aortic valve insufficiency. This study adequately demonstrates the safety and short-term reliable efficacy of transapical TAVR implantation of J-valves in patients with aortic valve insufficiency. Compared with the SAVR group, the TAVR group demonstrated potentially strong advantages in terms of faster postoperative recovery and bleeding, along with similar efficacy, although the incidence of perivalvular leakage was higher but predominantly mild, and the impact on short-term efficacy was not detected. It provides a rationale for further expansion of the indications for TAVR as the treatment of aortic regurgitation in the future.

## Figures and Tables

**Table 1:** Baseline data of TAVR and SAVR group before and after propensity score matching. (BMI, body mass index; STS Score, Society of Thoracic Surgeons Score; MI, myocardial infarction; CABG, coronary artery bypass grafting; PCI, percutaneous coronary intervention; COPD, chronic obstructive pulmonary disease; LVEF, left ventricular ejection fraction.).

Vabriable	Before Matching			After Matching		
	SAVR (*n* = 42)	TAVI (*n* = 69)	*p* Valve	SAVR (*n* = 16)	TAVI (*n* = 16)	*p* Valve
Male sex	34/42 (81%)	52/69 (75.4%)	0.49	11/16 (68.8%)	13/16 (81.3%)	0.41
Age(y)	69.52 ± 6.40	71.46 ± 7.92	0.18	67.94 ± 5.51	68.31 ± 5.10	0.84
STS Score (%)	1.48 ± 1.73	3.76 ± 3.93	<0.05	1.92 ± 2.64	1.89 ± 1.14	0.18
BMI (kg/m^2^)	21.90 ± 2.61	22.70 ± 3.15	0.18	23.10 ± 3.53	22.10 ± 2.03	0.36
NYHA class ≥ III	26/42 (61.9%)	53/69 (76.8%)	0.09	11/16 (68.8%)	13/16 (81.3%)	0.41
Hypertension	27/42 (64.3%)	48/69 (69.6%)	0.56	11/16 (68.8%)	12/16 (75.0%)	0.69
Diabetes	4/42 (9.5%)	9/69 (13.0%)	0.58	2/16 (12.5%)	1/16 (6.3%)	0.54
Smoker	22/42 (52.4%)	20/69 (29.0%)	<0.05	5/16 (31.3%)	7/16 (43.8%)	0.47
Alcohol	20/42 (47.6%)	25/69 (36.2%)	0.24	7/16 (43.8%)	8/16 (50.0%)	0.72
Coronary artery disease	17/42 (40.5%)	19/69 (27.5%)	0.16	5/16 (31.3%)	8/16 (50.0%)	0.28
Previous MI	0	0	-	0	0	-
Previous CABG	0	1/69 (1.4%)	0.44	0	0	-
Previous PCI	0	4/69 (5.8%)	0.11	0	0	-
Cerebral vascular disease	6/42 (14.3%)	6/69 (8.7%)	0.36	2/16 (12.5%)	2/16 (12.5%)	1.00
Peripheral vascular disease	3/42 (7.1%)	7/69 (10.1%)	0.59	1/16 (6.3%)	1/16 (6.3%)	1.00
COPD	4/42 (9.5%)	14/69 (20.3%)	0.07	4/16 (25.0%)	2/16 (12.5%)	0.37
Creatinine level > 2 mg/dL (177 μmol/liter)	1/42 (2.4%)	5/69 (7.2%)	0.27	0	0	-
Atrial fibrillation	2/42 (4.8%)	18/69 (26.1%)	<0.05	1/16 (6.3%)	0	0.31
Permanent pacemaker	3/42 (7.1%)	2/69 (2.9%)	0.30	1/16 (6.3%)	1/16 (6.3%)	1.00
LVEF(%)	57.30 ± 12.13	50.84 ± 12.38	<0.05	57.54 ± 12.07	55.21 ± 13.17	0.72

**Table 2:** Comparison of postoperative and 30-day follow-up results between the two groups before and after propensity score matching. ICU, intensive care unit; AF, atrial fibrillation; PPM, permanent pacemaker.

Outcome	Before Matching			After Matching		
	SAVR (*n* = 42)	TAVI (*n* = 68)	*p* Value	SAVR (*n* = 16)	TAVI (*n* = 16)	*p* Value
Hospital stay(day)	11.29 ± 3.73	11.41 ± 10.43	<0.05	12.63 ± 4.44	12.37 ± 13.79	<0.05
ICU stay(day)	5.05 ± 2.02	1.86 ± 5.78	<0.05	5.88 ± 2.60	3.50 ± 10.83	<0.05
Death from any cause	0	1/68 (1.5%)	0.43	0	0	-
Neurologic event	1/42 (2.4%)	2/68 (2.9%)	0.86	1/16 (6.3%)	0	0.31
Disabling stroke	0	2/68 (2.9%)	0.26	0	0	-
Nondisabling stroke	1/42 (2.4%)	0	0.20	1/16 (6.3%)	0	0.31
Perivalvular leakage	2/42 (4.8%)	23/68 (33.8%)	<0.05	0	6/16 (37.5%)	<0.05
≥Moderate	0	4/68 (5.9%)	0.11	0	1/16 (6.3%)	0.31
Major bleeding	11/42 (26.2%)	5/68 (7.4%)	<0.05	6/16 (37.5%)	1/16 (6.3%)	<0.05
Rehospitalization	1/42 (2.4%)	3/68 (4.4%)	0.58	0	0	-
New MI	0	0	-	0	0	-
New AF	1/42 (2.4%)	11/68 (16.2%)	<0.05	1/16 (6.3%)	2/16 (12.5%)	0.54
New PPM	0	5/68 (7.4%)	0.07	0	1/16 (6.3%)	0.31
Endocarditis	1/42 (2.4%)	0	0.20	0	0	-

## Data Availability

Not applicable.
